# Human Amniotic Epithelial Stem Cells Alleviate Autoimmune Premature Ovarian Insufficiency in Mice by Targeting Granulosa Cells via AKT/ERK Pathways

**DOI:** 10.1007/s12015-024-10745-z

**Published:** 2024-06-04

**Authors:** Xiaohang Ye, Yifeng Lin, Yanyun Ying, Xuezhi Shen, Feida Ni, Feixia Wang, Jianpeng Chen, Wei Zhao, Xiaoming Yu, Dan Zhang, Yifeng Liu

**Affiliations:** 1 Key Laboratory of Reproductive Genetics (Ministry of Education) and Department of Reproductive Endocrinology, Women’s Hospital, Zhejiang University School of Medicine, Zhejiang, 310006 China; 2 Zhejiang Provincial Clinical Research Center for Child Health, Hangzhou, 310006 China

**Keywords:** Human amniotic epithelial stem cells, Autoimmune premature ovarian insufficiency, Granulosa cells, AKT, ERK

## Abstract

Autoimmune factors play an important role in premature ovarian insufficiency (POI). Human amniotic epithelial stem cells (hAESCs) have recently shown promising treatment effects on chemotherapy-induced POI. However, the therapeutic efficacy and underlying mechanisms of hAESCs in autoimmune POI remain to be investigated. In this study, we showed for the first time that intravenous transplantation of hAESCs could reside in the ovary of zona pellucida 3 peptide (pZP3) induced autoimmune POI mice model for at least 4 weeks. hAESCs could improve ovarian function and fertility, alleviate inflammation and reduce apoptosis of granulosa cells (GCs) in autoimmune POI mice. The transcriptome analysis of mice ovaries and in vitro co-cultivation experiments suggest that activation of the AKT and ERK pathways may be the key mechanism in the therapeutic effect of hAESCs. Our work provides the theoretical and experimental foundation for optimizing the administration of hAESCs, as well as the clinical application of hAESCs in autoimmune POI patients.

**Graphical Abstract:**

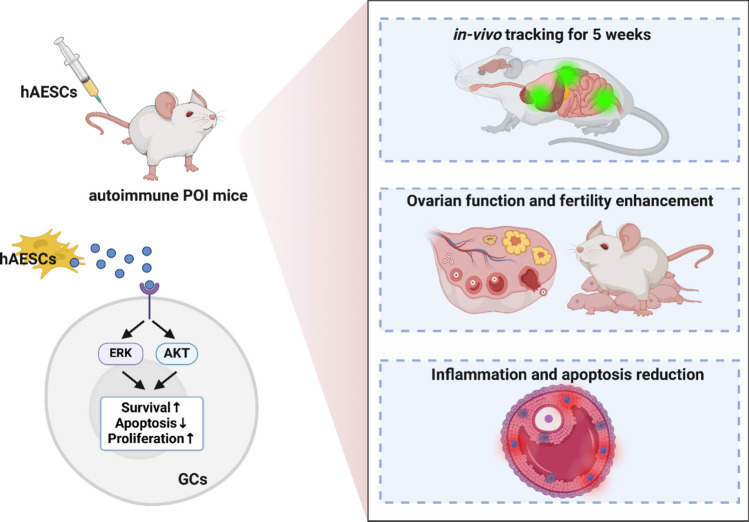

**Supplementary Information:**

The online version contains supplementary material available at 10.1007/s12015-024-10745-z.

## Introduction

Premature ovarian insufficiency (POI) is a clinical syndrome defined by the loss of ovarian activity and high serum level of follicle-stimulating hormone (FSH) before the age of 40 which has affected 1 ~ 5% of women worldwide. The etiology of POI has been linked to genetic defects, iatrogenic damage, immune dysfunction, virus infection and environmental factors [1, 2]. It is estimated that 10 ~ 55% of patients with POI have a concomitant autoimmune disease, especially autoimmune thyroiditis, Addison’s disease and type 1 diabetes mellitus [3] with autoimmune factor believed to participate in 4 ~ 30% of POI [4]. Patients with positive thyroid antibodies have reduced number of retrieved oocytes during assisted reproduction, with immunological microenvironment alterations in follicles [5]. Up to 10 ~ 20% of patients with Addison’s disease and 2.5% of patients with diabetes mellitus will develop POI [3]. Moreover, the presence of ovarian autoantibodies and histological confirmation of lymphocytic oophoritis further suggest the potential relevance between autoimmunity and POI [6]. Nowadays, immunizing BALB/c mice with zona pellucida 3 peptide (pZP3) could induce high levels of anti-zona pellucida antibodies, leading to a rapid reduction and atresia of growing follicles, which was the most widely used autoimmune POI animal model [7].

Patients with POI may experience various health concerns, including estrogen-deficiency symptoms, infertility, mental problems, osteoporosis, increased risk of cardiovascular disease, reduced life expectancy and so on [2, 8–10]. Considering that many POI complications are related to estrogen deficiency, hormone replacement therapy (HRT) currently stands as the most commonly used treatment for POI which can mimic normal ovarian endocrine function [8, 11]. However, HRT exhibits little efficacy on enhancing fertility and ovarian activity, and brings with increased risk of breast cancer, venous thromboembolism and cardiovascular diseases [12, 13]. Besides, patients with POI show a high discontinuation rate (35% ~ 64.2%) and poor compliance with HRT due to its long-term use [14, 15]. Therefore, there is a pressing need for new methods to improve the ovarian function and long-term outcomes of patients with POI.

Recently, stem cell therapy has attracted great interest in the treatment of POI, due to its considerable therapeutic potential and outstanding performance in regenerative medicine [16]. Human amniotic epithelial stem cells (hAESCs), derived from placentae, possess distinct advantages over other therapeutic stem cells including ample availability, little ethical controversies, minimal DNA damage and low tumorigenicity [17, 18]. In addition, hAESCs have been proven to mediate anti-inflammation process through both cytokine secretion and direct cell-to-cell contact [17]. Based on their damage repair capabilities and immunomodulatory properties, hAESCs have been considered as a promising treatment tool for various conditions, particularly for immune-mediated diseases, including autoimmune uveitis [19], Crohn's disease [20], psoriasis [21], hashimoto’s thyroiditis and systemic lupus erythematosus [22]. Some studies have demonstrated that hAESCs can restore ovarian function in chemotherapy-induced POI model [23–27]. However, little attention has been paid to the effect of hAESCs on autoimmune POI. Considering their powerful regenerative repair ability and anti-inflammatory properties, we hypothesize that hAESCs could also perform well in the treatment of autoimmune POI.

This current study aims to investigate the effect and underlying mechanisms of hAESCs on the pZP3-induced autoimmune POI mice model.

## Materials and Methods

### Experimental Design, Animals, and Management

Female BALB/c mice aged 6–8 weeks (SLAC, China) were housed in the laboratory animal center of Zhejiang University under controlled conditions (21–24 °C, relative humidity 40–60%, 12 h light/dark cycle), with free access to food and water. The mice were randomly divided into three groups: the control mice (the Ctrl group), the autoimmune POI mice (the POI group) and the hAESCs-treatment mice (the hAESCs group). To establish the autoimmune POI model, mice would receive pZP3 immunizations twice, as previously described [28]. Briefly, we dissolved the peptide of murine ZP3 330–342 (NSSSSQFQIHGPR) (Yuantai, China) at 1 mg/mL in sterilized double-distilled water and used Vibra-Cell™ Ultrasonic Liquid Processor (SONICS, US) to emulsified the pZP3 solution with equal volume of complete Freund's adjuvant (CFA; Sigma-Aldrich, Germany). Subsequently, subcutaneous and foot pad injection were administered with 150ul CFA-pZP3 emulsion per mouse. The same process was performed for booster immunization with the pZP3 solution and incomplete Freund's adjuvant (Sigma-Aldrich, Germany) 14 days after primary immunization. Vaginal smear analysis was applied to determine the success of model establishment: the POI mice were expected to show irregular estrus cycles. 28 days after primary immunization, the hAESCs group would receive 1 × 10^6^ hAESCs (iCell Biotechnology, China) via vein tail injection. Except mice used in in vivo tracking and mating experiments, the majority of mice were euthanized around 4 weeks after hAESCs transplantation at diestrum, and samples were collected for further experiments.

All experimental protocols in this study were performed following the guidelines for the humane use of laboratory animals and were approved by the Ethics Committee of the Laboratory Animal Center of Zhejiang University (ZJU20230493 authorization).

### In Vivo Tracing of hAESCs

hAESCs were labeled by the cell membrane fluorescent probe, CM-DiD (λEx/λEm = 644/663 nm) or CM-DiR (λEx/λEm = 748/780 nm) (US Everbright, China), according to the manufacturer’s instructions. hAESCs were suspended in PBS to a density of 10^6^ cells/mL and then were mixed with 1 mM CM-DiD or CM-DiR to a final concentration of 1 μM. After incubation for 20 min at 37 °C, the cells were washed twice by PBS. The whole body and the isolated organs of POI mice injected with CM-DiR labeled hAESCs were used for imaging under the in vivo imaging system (IVIS) at set time. Organs from POI mice injected with CM-DiD labeled hAESCs were used for the frozen section. Slides of 5 µm thick were dyed with DAPI (Beyotime Biotechnology, China) and observed under Olympus IX81-FV1000 fluorescence microscope.

### Estrous Cycle Analysis

Vaginal smear analysis was performed at 13:00–14:00 every day for 20 days. 20 μL PBS was gently added into the mouse vagina using a pipette and then the liquid was smeared on the glass slide. After air drying, the samples were stained with methylthionine chloride. The stages of the estrous cycle were estimated under the light microscope as previously described [1].

### Enzyme-Linked Immunosorbent Assay (ELISA)

Blood samples were collected via eyeball extracting, coagulated at room temperature for 1 h and then centrifuged at 1500 r/ minutes for 20 min. Subsequently, serum was isolated from the upper layer. Following the manufacturer's instructions of the ELISA kits (Mei-mian, China), serum concentrations of FSH, E2 and anti-müllerian hormone (AMH) were measured.

### Fertility Test

Female mice from three groups were mated with fertile males aged 8–10 weeks (female: male = 1:1) 2 weeks after stem cell transplantation. They were allowed to undergo consecutive natural pregnancies up to 3 times within 12 weeks and litter sizes were recorded.

### Histomorphology Analysis of Ovaries

Ovaries collected 4 weeks after hAESCs transplantation were fixed with 4% paraformaldehyde, dehydrated with graded alcohol and xylene, and embedded in paraffin for subsequent analysis. To analyze the ovarian morphology and follicle counts, the whole ovaries were cut into 5 μm serial sections. After hematoxylin–Eosin (H&E) staining under standard methods, the ovarian morphology was observed and imaged by an optical microscope (OLYMPUS VS200, Japan) and the number of follicles was counted. The follicles were classified as primordial, primary, secondary and antral follicles, as previously described [1]. Primordial and primary follicles were counted every 5th section and multiplied the raw count by 5 as the final count. While the secondary and antral follicles were counted in every section. Only follicles containing a nucleated oocyte were counted to avoid repetition.

### Immunohistochemistry Analysis

Immunohistochemistry staining was performed following a standard protocol, as previously described [29], with primary antibodies against anti-CD45 (70,257, CST, USA), anti-TNF-α (ER1919-22, Huabio, China) and anti-Caspase3 (66,470–2-Ig, Proteintech, China). Immunohistochemical scores (IHS) of the sections were independently evaluated by 2 observers. For evaluation, five randomly selected fields (200X) were assessed for each section. The scoring criteria were the same as previously described [30].

### Transmission Electron Microscope (TEM) Imaging

The fresh ovaries were fixed in 2.5% pentanediol buffer for 2 h at room temperature and then fixed overnight at 4 ℃. Then the samples were washed twice with PBS and were fixed with 1% osmic acid for 1 h. Subsequently, the samples were washed twice with double-distilled water and were then fixed with 2% uranyl acetate for 30 min. Then the samples were dehydrated in 50%, 70%, 90%, 100% ethanol and 100% acetone, each for 15 min. Then the samples were embedded at room temperature with 1:1 embedding agent–acetone and 3:1 embedding agent–acetone, each for 2 h. At last, the samples were transferred to 100% embedding agent, embedded at 37 °C and polymerized. Ultrathin sectioning was prepared by Ultramicrotome Leica EM UC7 (Leica, US) and observed by Tecnai G2 Spirit TWIN TEM (FEI, US) operating at 120kv.

### RNA Sequencing and Data Analysis

Libraries for mRNA sequencing and bioinformatics analysis were constructed by LC-Bio Technology CO as previously described [31]. Total RNA was isolated and purified using TRIzol reagent (Invitrogen, USA) following the manufacturer's procedure. Each sample contains two ovaries. Then we performed the 2 × 150 bp paired-end sequencing (PE150) on Illumina Novaseq™ 6000 (LC-Bio, China) following the vendor's recommended protocol. After removal of adaptors, junk and low-quality sequences, the clean data were mapped to reference genome using HISAT2 (v2.0.4). StringTie (v1.3.4) and gffcompare (v0.9.8) was further used to quantify expression level for mRNAs by calculating FPKM. The significant differentially expressed genes (DEGs) were identified under the criteria of |fold change|> 1.75 and adjusted p value < 0.05 by R package DESeq2. Hierarchical cluster analysis, volcano plot, venn diagram and Kyoto Encyclopedia of Genes and Genomes (KEGG) pathway enrichment analysis were performed using the OmicStudio tools (https://www.omicstudio.cn/tool). Protein–protein interaction (PPI) predictions were performed using the STRING database and all interactions were filtered with a minimum confidence score of 0.4. The PPI network diagram was plotted using Cytoscape (v3.8.2).

### Quantitative Real‐Time Polymerase Chain Reaction (qRT-PCR)

qRT-PCR was performed using a HiScript II U^+^ One Step qRT-PCR Probe Kit (Vzayme, China) on a LightCycler 480 (Roche, Switzerland) according to the manufacturer’s protocol. GAPDH was used as an endogenous reference for mRNA normalization. The PCR primer sequences were listed in Additional file 1: Table S1.

### Cell Culture

The human ovarian granulosa-like tumor cell line KGN (CL-0603, Procell, China) were cultured in DMEM/F12 (HyClone, USA) containing 10% fetal bovine serum (Gibco, USA) and 1% penicillin plus streptomycin (Procell, China). In addition to the above ingredients, the medium of hAESCs needed extra 10 ng/mL EGF (PeproTech, USA). Passaging would be performed when the cell confluence reached 80%-90%. Only early passages of hAESCs (passages 0–1) were used in the following experiments. All cells were cultured in a humidified incubator at 37 °C with 5% CO_2_.

### Preparation of Conditioned Medium of hAESCs (hAESCs-CM)

When hAESCs reached 80–90% confluence in the complete medium with EGF, the medium was replaced with complete medium without EGF. Following 24 h of culture, the conditioned medium (CM) was collected. The hAESCs-CM was stored under –80 °C for subsequent experiments. The complete medium without EGF was used to dilute hAESCs-CM to 25%—50% for the co-cultivation experiment.

### Co-Cultivation of KGN and hAESCs

KGN (1.5 × 10^4^ cells/well for 24-well plates or 7 × 10^4^ cells/well for 6-well plates) were cultured in lower chamber of transwells (4-μm, 6/24-well insert; Corning, USA). hAESCs (1.2 × 10^4^ cells/well for 24-well plates or 5 × 10^4^ cells/well for 6-well plates) were cultured in upper chamber of transwells. After 24 h for cell adhesion, co-cultivation were conducted with both medium replaced by complete medium without EGF. Then different concentrations of H_2_O_2_, LY294002 (S1737, Beyotime Biotechnology, China) and U0126 (HY-12031, MedChemExpress, US) were added to the systems for 24, 48 or 72 h.

### Cell Counting kit-8 (CCK8) Assay

KGN were cultured in 96-well plates (1 × 10^3^ cells/well) with or without H_2_O_2_, inhibitors and hAESCs-CM at set concentrations according to study design. 10 μL of CCK8 solution (Biosharp, China) was added to each well and incubated at cellular incubation conditions for 2 h. The optical density was measured at 450 nm using spectrophotometry (Bio-Rad, USA). The relative cell viability = [OD (test)-OD (culture medium)]/ [OD (the Crtl group)-OD (culture medium)].

### Ethynyl-2-Deoxyuridine (EdU) Assay

Proliferation assay of KGN in 24-well transwells was performed using EdU Imaging Kits (US Everbright, China), according to the manufacturer's instructions. Images from five random fields each well were observed by the Olympus IX81-FV1000 fluorescence microscope. The percentage of EdU-positive cells indicated cell proliferation ability.

### Flow Cytometry

Cell apoptosis was measured by Annexin V-FITC/PI apoptosis detection kit (BD Biosciences, USA), according to the manufacturer's instructions. In brief, adherent and suspension KGN were both harvested, washed and last suspended in 1 × Binding Buffer. Then, 5μL of FITC Annexin V and 5μL of PI were added to the cell suspension and incubated for 25 min in the dark at room temperature. All samples were analyzed by the CytoFlex S flow cytometry (Beckman Coulter, US).

### Western Blot Analysis

Proteins were extracted from KGN using RIPA buffer (Biosharp, China) with 1 × phenylmethanesulfonyl fluoride (Biosharp, China) and 1 × protease inhibitor cocktail (MCE, USA) and denatured by 5 × SDS-PAGE loading buffer (CWBIO, China). Then they were separated by SDS-PAGE and transferred to nitrocellulose membranes (HATF00010, Millipore, Germany). After being blocked with 5% bovine serum albumin for 60 min at room temperature, the membranes were incubated with primary antibodies overnight at 4 °C. After being washed thrice with PBS with 0.1% Tween-20 (PBST), the membranes were incubated with secondary antibodies for 60 min at room temperature and then washed thrice with PBST. Finally, the membranes were captured by ChemiDoc Touch Imaging System (Bio-Rad, USA). The following antibodies were used: anti-p-AKT (9275, CST, USA), anti-AKT (4821S, CST, USA), anti-p-Erk1/2 (4370, CST, USA), anti-Erk1/2 (4695, CST, USA), anti-β-Actin (3700, CST, USA), anti-BAX (ET1603-34, HuaBio, China), anti-BCL2(ER1802-97, HuaBio, China), anti-Caspase3 (66,470–2-Ig, Proteintech, USA), Goat anti-mouse fluorescent antibody (926–68,020, LI-COR, USA) and Goat anti-rabbit fluorescent antibody (926–32,211, LI-COR, USA).

### Statistical Analysis

Data were collected from at least three independent experiments and were expressed as mean ± standard deviation (SD). One-way ANOVA test followed by Student–Newman–Keuls analysis was used for multiple comparisons. The level of statistical significance was defined as *P* < 0.05. All data analysis were performed with GraphPad Prism 8.0.

## Results

### In vivo Tracking of hAESCs in the Autoimmune POI Mice

**Fig. 1:**
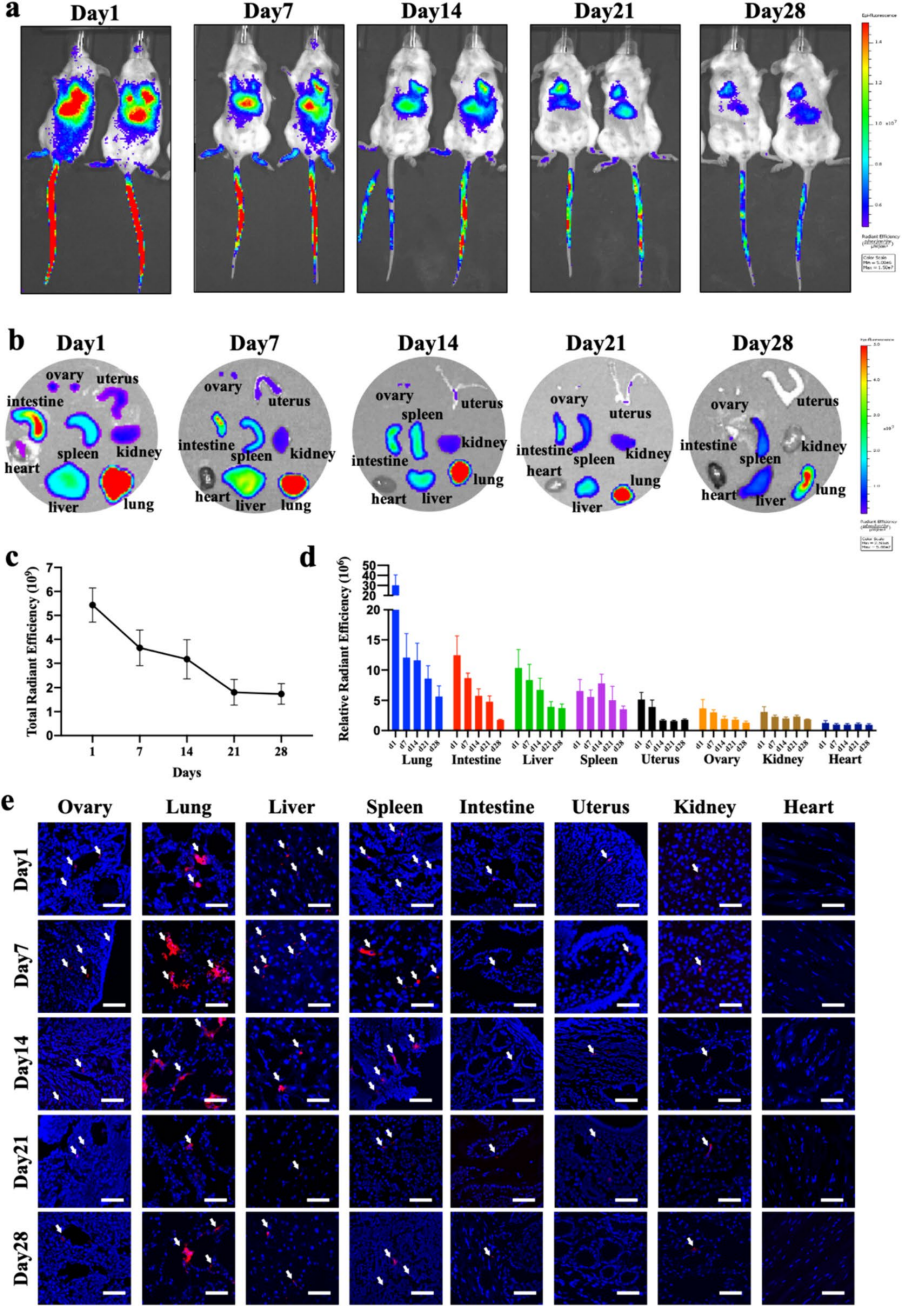
In vivo tracking of hAESCs in autoimmune POI mice. **a**. Whole-body imaging of autoimmune POI mice at 1, 7, 14, 21, and 28 days after DiR-labeled hAESCs transplantation under the IVIS system. **b**. Isolated-organ imaging of autoimmune POI mice at 1, 7, 14, 21, and 28 days after DiR-labeled hAESCs transplantation under the IVIS system. **c**. The total radiant efficiency of the whole mice over time (n = 5). **d**. The relative radiant efficiency of isolated-organs over time (n = 5). **e**. Frozen sections of isolated-organs at 1, 7, 14, 21, and 28 days after DiD-labeled hAESCs (red) transplantation, in which the nuclei of cells were labeled with DAPI (blue) (bar = 50 μM). The arrows indicate DiD-labeled hAESCs. Data were presented as the means ± SD

After transplanting 1 × 10^6^ DiR-labeled hAESCs via tail vein injection, we detected the fluorescence signal of the whole mouse at the set times (1, 7, 14, 21, and 28 days). Although the fluorescence intensity from DiR-labeled hAESCs decreases with time, the fluorescence signal could be detected in multiple regions of the thoracic and abdominal cavities (Fig. 1a and c). Furthermore, isolated-organ imaging showed that hAESCs mostly enriched in the lungs, followed by the intestines, liver and spleen. At the same time, hAESCs could also be detected in the uterus, ovaries and kidneys, but seemed unable to enter the heart tissue (Fig. 1b and d).

Consistent with the results of whole-body imaging and organ imaging, DiD-labeled hAESCs mainly resided in the lungs according to the frozen section (Fig. 1e). Although not many hAESCs were enriched in the ovary, they could reach the granulosa cell (GC) layer of follicles and could still be detected 28 days after transplantation.

### hAESCs Restored Ovarian Function of Autoimmune POI Mice

**Fig. 2:**
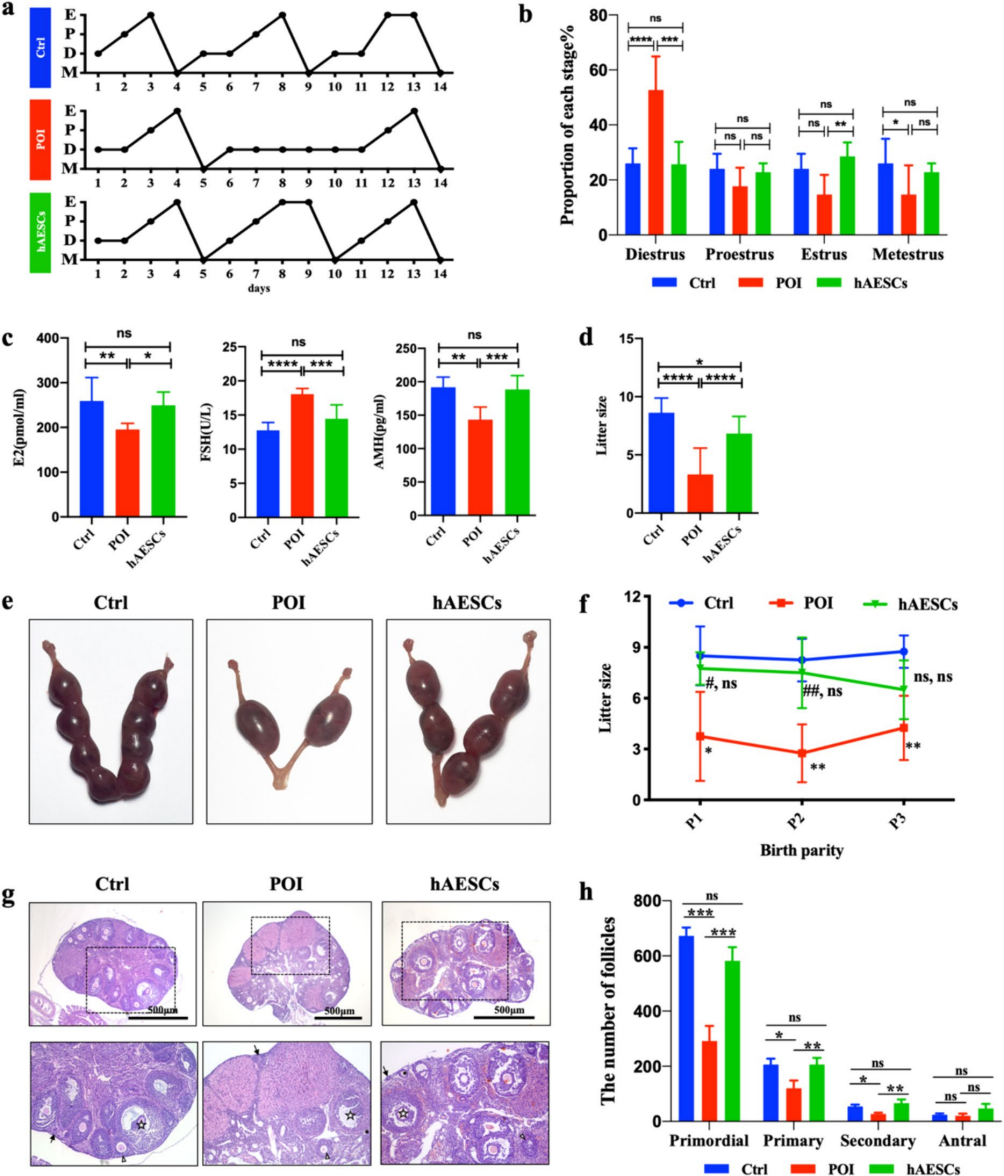
hAESCs transplantation restored autoimmune POI. **a**. Representative estrous cycle patterns in three groups (E, estrus; P, proestrus; D, diestrus; M, metestrus). **b**. The percentage of four stages in three groups was calculated after hAESCs transplantation for 20 days (n = 5). **c**. The serum level of FSH (n = 6), E2 (n = 7) and AMH (n = 6) at 2 weeks after hAESCs therapy. **d**. Litter sizes of three groups during 12 weeks of consecutive births (n = 4). **e**. Representative images of embryo implantation in three groups. **f**. Litter sizes of three groups during 3 consecutive births (n = 4). **g**. Representative H&E staining of ovaries from three groups, scale bar = 500 μm. The arrows indicate primordial follicles, the triangles indicate primary follicles, the asterisks indicate secondary follicles, the pentagrams indicate antral follicles. **h**. The follicle numbers of four-stages in three groups (n = 3). Data were presented as the means ± SD. ns: *P* > 0.05, *P < 0.05, **P < 0.01, ***P < 0.001, ****P < 0.0001

As shown in Fig. 2a and b, the estrous cycle of the Ctrl group repeats every 4–5 days. However, the diestrus was remarkably prolonged in autoimmune POI mice, leading to relatively infrequent complete estrous cycles. Compared with the POI group, the diestrus in the hAESCs group was shortened with no significant difference from the Ctrl group.

In addition, serum hormone levels were detected to determine the effect of hAESCs on ovarian endocrine levels of autoimmune POI. Compared with the Ctrl group, the POI group showed significantly higher levels of FSH and lower levels of E2 and AMH (Fig. 2c). While hAESCs therapy could significantly reduce the levels of FSH and increase the levels of E2 and AMH to normal conditions (Fig. 2c).

The POI group showed markedly subfertility compared with the Ctrl group, whereas the hAESCs treatment led to significantly increased litter size although there was still a certain gap compared with the fertility of healthy mice (Fig. 2d and e). Besides, we found that during three consecutive births, the litter sizes in each birth of the POI group were all significantly lower than that of the Ctrl group (Fig. 2f). The litter sizes of the hAESCs group were significantly improved in the first two births, with no significant difference from the Ctrl group, and then decreased in the third births.

H&E staining was performed to explore the effect of hAESCs on ovarian structure in autoimmune POI mice. As shown in Fig. 2g and h, ovaries of the POI group had less primordial, primary and secondary follicles than the Ctrl group. While hAESCs treatment inhibited the decline in the numbers of follicles of these three stages, with no difference from that of the Ctrl group. Moreover, the number of antral follicles also showed an increasing trend after hAESCs therapy compared with the POI mice, but did not reach a significant difference.

These results demonstrated that hAESCs transplantation could significantly remedy the ovarian function of POI mice by stabilizing estrus cycle, restoring hormonal levels, promoting fertility and increasing follicular numbers.

### hAESCs Alleviated the Oophoritis and GC Apoptosis in Autoimmune POI

**Fig. 3:**
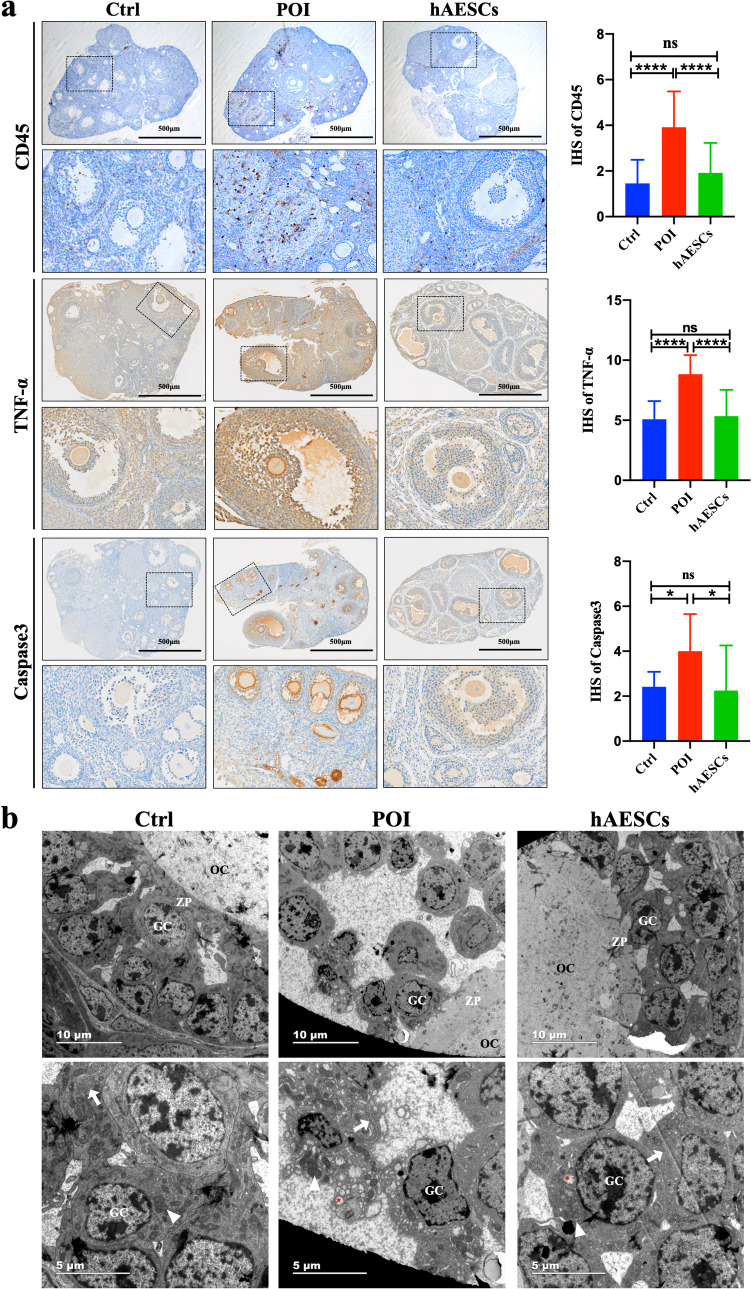
hAESCs alleviated the oophoritis and GC apoptosis. **a**. Representative immunohistochemical staining and average IHS of CD45, TNF-α and Caspase3 in ovaries of three groups. Scale bar = 500 μm. **b**. Representative TEM Images of GCs in the ovarian follicles of three groups, Scale bar = 10 or 5 μm. OC: oocyte, GC: granulosa cell, ZP: zona pellucida. The arrows indicate the endoplasmic reticulum, the triangles indicate the mitochondria, the red asterisks indicate the vacuoles. Data were presented as the means ± SD. ns: *P* > 0.05, *P < 0.05, ****P < 0.0001

Previous studies have reported that, histopathological evidence of autoimmune POI is characterized by the presence of intense lymphocytic infiltration in the ovaries with a characteristic sparing of primordial follicles [6]. Therefore, CD45 (leukocyte common antigen) immunohistochemical staining was used to access inflammatory cell infiltration in ovaries of the three groups. As shown in Fig. 3a, CD45-positive cell infiltration in the ovaries of the POI group was significantly increased compared with the Ctrl group, while the ovaries of the hAESCs group showed less CD45-positive cell infiltration. In addition, the expression of pro-inflammatory cytokine, TNF-α, was also tested in the ovaries among three groups. Consistent with the results of CD45, the expression level of TNF-α in the ovaries of the POI group was significantly increased compared to the Ctrl group, while the hAESCs transplantation significantly reduced the TNF-α level.

Caspase3 immunohistochemistry was carried out to evaluate apoptosis level of ovaries. As shown in Fig. 3a, the expression level of Caspase3 in the ovaries of the POI group was significantly higher than that of the Ctrl group, and were mainly distributed in the atretic follicles and the layer of GCs. After hAESCs transplantation, the expression level of Caspase3 was significantly decreased.

To further evaluate the effect of hAESCs on GCs, transmission electron microscopy (TEM) was used to observe the ultrastructure of the layers of GCs in each group. As shown in Fig. 3b, in the Ctrl group, the GCs were rich in mitochondria with clear mitochondrial cristae, and the shape of the endoplasmic reticulum was regular. Whereas, the GCs from the POI group showed significant apoptotic characteristics including abundant vacuoles, dilated endoplasmic reticulum, and swollen mitochondria with blurred mitochondrial cristae. Additionally, the intercellular space between GCs was enlarged. hAESCs treatment significantly improved the ultrastructure of GCs, with the cell arrangement as well as the morphology of the organelles significantly restored and the number of vacuoles significantly reduced.

### RNA-seq Analysis of Transcriptome in Mouse Ovaries After hAESCs Transplantation

**Fig. 4:**
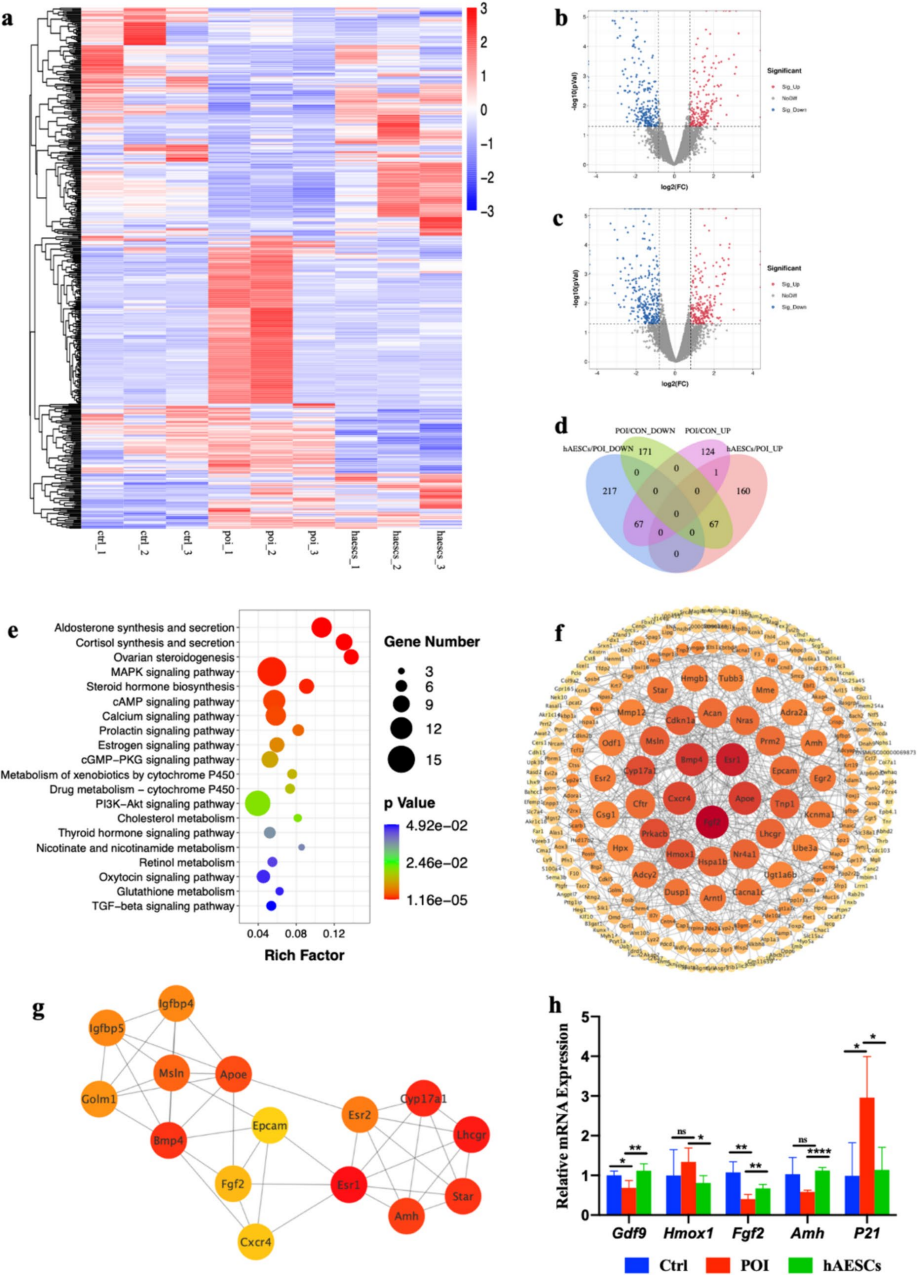
RNA-seq analysis to reveal the effect of hAESCs transplantation. **a**. Heatmap of the DEGs in the 9 ovarian samples from three groups. The color key from blue to red indicates the relative gene expression level from low to high, respectively. b-c. Volcano plots of total gene expression profiles of pairwise comparisons. The Ctrl group vs. the POI group (**b**); the POI group vs. the hAESCs group (**c**). The red and blue dots correspond to significantly up-regulated (Sig_Up) and significantly down-regulated (Sig_Down) genes, respectively. **d**. Venn diagrams of the DEGs between pairwise comparisons. **e**. Significantly enriched KEGG pathways of candidate DEGs. **f**. Major PPI network of candidate DEGs. The color and the area of a certain node indicated the degree of interaction. **g**. Subnetwork analysis by CytoHubba module showed the 15 highest-score nodes. **h**. qRT-PCR validation of relative expression of the candidate DEGs. Data were presented as the means ± SD. ns: *P* > 0.05, *P < 0.05, **P < 0.01, ****P < 0.0001

To further understand the effect of hAESCs on the expression profile of ovaries among the three groups, RNA-seq analysis was performed. Hierarchical cluster analysis showed the expression patterns of differentially expressed genes (DEGs) (Fig. 4a). In addition to three-group comparison, we also performed pairwise comparisons between the Ctrl group and the POI group, as well as between the POI group and the hAESCs group (Fig. 4b and c). Furthermore, Venn diagram was used to screen candidate DEGs, as shown in Fig. 4d. 192 up-regulated DEGs and 238 down-regulated DEGs were identified in comparison between the Ctrl and autoimmune POI groups. After hAESCs transplantation, a total of 134 POI-related DEGs were reversed (67 genes induced by autoimmune POI were suppressed, and 67 genes suppressed in autoimmune POI were up-regulated). Besides, hAESCs therapy additionally induced 160 genes and suppressed 217 genes.

We speculated that DEGs reversed and additional induced or suppressed after hAESCs therapy, 511 DEGs in total, might related to the mechanism of the hAESCs therapy. Therefore, these candidate DEGs were summed up for subsequent functional enrichment analysis. As shown in Fig. 4e, KEGG enrichment analysis indicated that these 511 DEGs were mainly enriched in ovarian steroidogenesis, MAPK pathway, cAMP pathway, estrogen pathway, cGMP-PKG pathway, PI3K-Akt pathway and TGF-beta pathway.

Protein–protein interaction (PPI) network of candidate DEGs was established. As shown in Fig. 4f the main PPI network was with the most nodes and the most boundaries, which contains 277 nodes and 589 edges. The degree of each node was calculated based on the number of its connections to other nodes. The 10 highest-degree nodes were screened as central genes: Fgf2, Esr1, Bmp4, Apoe, Cxcr4, Prkacb, Cyp17a1, Hmox1, Tnp1, Msln and Cdkn1a (P21). Furthermore, the subnetwork analysis by CytoHubba module demonstrated the 15 highest-score nodes as hub genes: Esr1, Lhcgr, Cyp17a1, Star, Bmp4, Amh, Apoe, Msln, Esr2, Igfbp5, Igfbp4, Golm1, Fgf2, Cxcr4 and Epcam (Fig. 4g).

Based on the above analysis, we verified the mRNA expression level of several candidate DEGs in three groups, including Gdf9, Hmox1, Fgf2, Amh and P21, which were of great importance for ovarian function and follicular development (Fig. 4h). The consistency between the RNA-seq and the qRT-PCR results confirmed the reliability of RNA-seq.

### hAESCs Promoted Proliferation and Inhibited Apoptosis of H_2_O_2_‐Induced Inflammation in KGN by Activation of AKT and ERK Pathway

As mentioned above, most of the apoptosis in the ovaries of the autoimmune POI group occurred in GCs. KGN were human granulosa-like cell line from a steroidogenic human granulosa cell tumor. H_2_O_2_ stimulation was used to build a cell model of inflammatory injury [32, 33]. hAESCs-CM or hAESCs co-cultivation was applied to further verify the repair capacity of hAESCs and to find out the potential mechanisms.

**Fig. 5:**
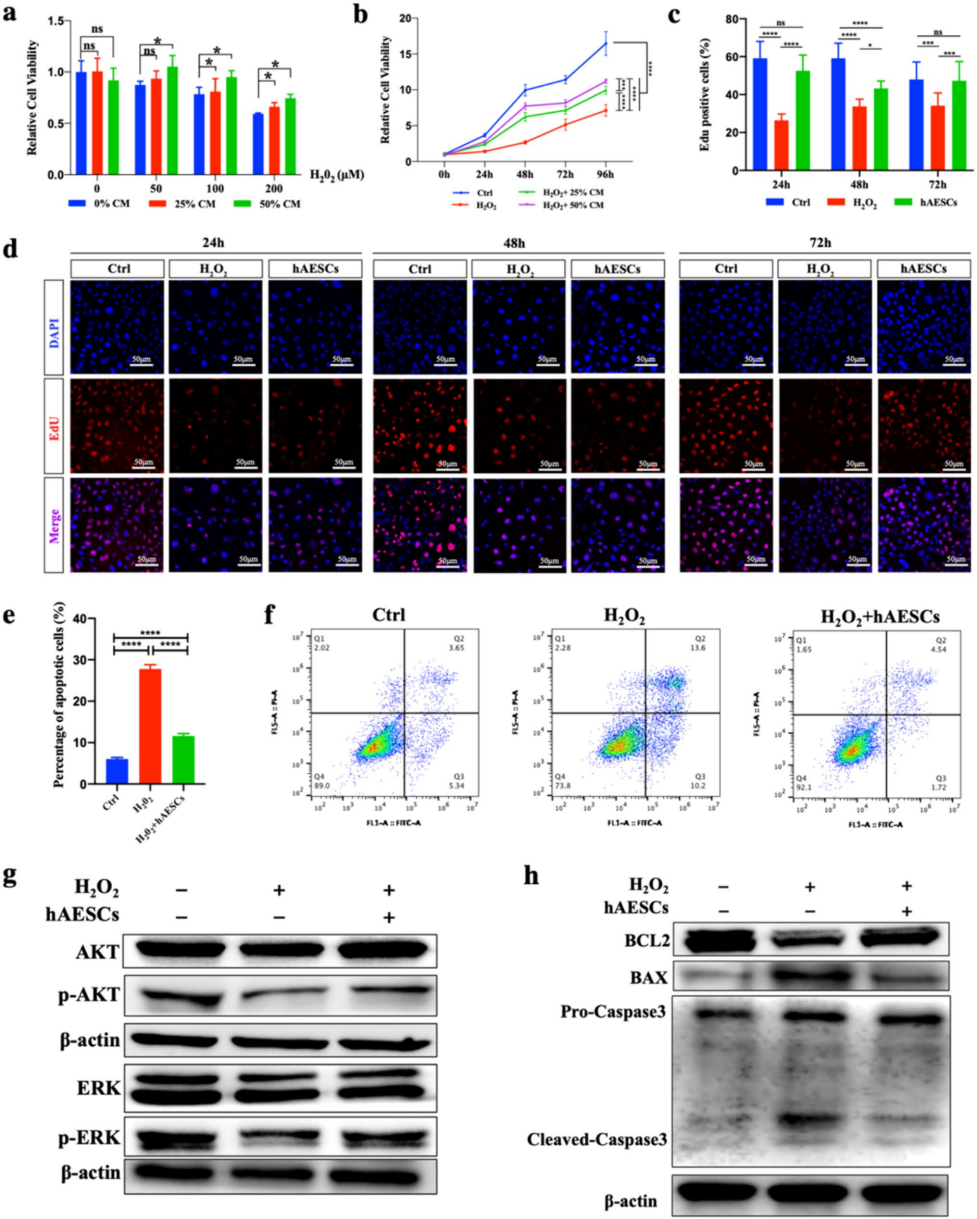
The effects of hAESCs-CM and hAESCs on H_2_O_2_ injured KGN. **a**. The viability of KGN under different concentrations of H_2_O_2_ with or without hAESCs-CM for 24 h. **b**. The viability of 200 μM H_2_O_2_ treated KGN with or without hAESCs-CM for 24, 48 and 72 h. **c**. Percent of EdU positive cells in control KGN and 200 μM H_2_O_2_ treated KGN co-cultured with or without hAESCs for 24, 48 and 72 h. **d**. Immunofluorescence staining images of DAPI (blue) and EdU (red) of three groups of KGN after co-cultivation for 24, 48 and 72 h. Scale bar = 50 μm. **e**. Percent of apoptosis cells (Annexin V-FITC positive cells) in three groups of KGN after co-cultivation for 24 h. **f**. Flow-cytometric analysis of Annexin V-FITC/PI of three groups of KGN after co-cultivation for 24 h. **g**. Western blot of AKT, p-AKT, ERK, p-ERK and β-actin in three groups of KGN. **h**. Western blot of BCL2, BAX, Pro-Caspase3, Cleaved-Caspase3 and β-actin in three groups of KGN. Data were presented as the means ± SD. ns: *P* > 0.05, *P < 0.05, **P < 0.01, ***P < 0.001, ****P < 0.0001

First, we treated KGN with H_2_O_2_ at different concentrations (0, 50, 100, 200 μM) with the hAESCs-CM accounted for 0, 25% or 50% of the total culture medium. CCK8 detection was performed 24 h after treatment. As shown in Fig. 5a, it was found that 25% hAESCs-CM could significantly increase the survival of KGN under 100 and 200 μM H_2_O_2_ treatment, and 50% hAESCs-CM could significantly increase the survival of KGN under 50, 100, 200 μM H_2_O_2_. Thus, 200 μM H_2_O_2_ was selected for subsequent cell experiments. As shown in Fig. 5b, the cell viability of KGN from different groups at multiple time points was also detected. Consistently, 25% and 50% hAESCs-CM could significantly increase the cell viability of 200 μM H_2_O_2_ treated KGN for 96 h. The therapeutic effect of 50% hAESCs-CM is better than that of 25%, although neither could completely reverse the damage of H_2_O_2_.

Furthermore, transwells were used to co-culture H_2_O_2_ treated KGN with hAESCs for 24, 48, and 72 h, before EdU exposure. The rate of EdU-positive cells indicated the cell proliferative capacity. As shown in Fig. 5c and d, H2O2 significantly impaired the proliferation ability of KGN, while hAESCs co-cultivation could significantly improve its impaired proliferation ability.

The apoptosis levels of KGN in each group were also detected after co-cultured with hAESCs for 24 h. As Annexin V-FITC positive cells represented apoptosis cells, H_2_O_2_ significantly increased the apoptosis of KGN, while hAESCs co-cultivation significantly reduced the apoptosis of KGN caused by H_2_O_2_ (Fig. 5e and f).

Given the results of KEGG analysis and the importance of AKT and ERK pathway in ovarian function, the activation of AKT and ERK and the expression of apoptosis-related molecules were detected in H_2_O_2_ treated KGN with or without hAESCs co-cultivation. As shown in Fig. 5g, H2O2 inhibited the phosphorylation of AKT and ERK while hAESCs treatment could activate the phosphorylation of AKT and ERK. Besides, H_2_O_2_ stimulation significantly increased the expression of pro-apoptosis markers of BAX and Cleaved-Caspase3, and reduced the expression of anti-apoptotic marker of BCL2 which could be reversed by hAESCs treatment (Fig. 5h).

### The effects of hAESCs on H_2_O_2_ Injured KGN could be Reversed by the Inhibitors of AKT and ERK Pathways

The inhibitors of the AKT (LY294002) and ERK (U0126) pathways were used to further confirm the role of AKT and ERK pathways in hAESCs treatment.

**Fig. 6:**
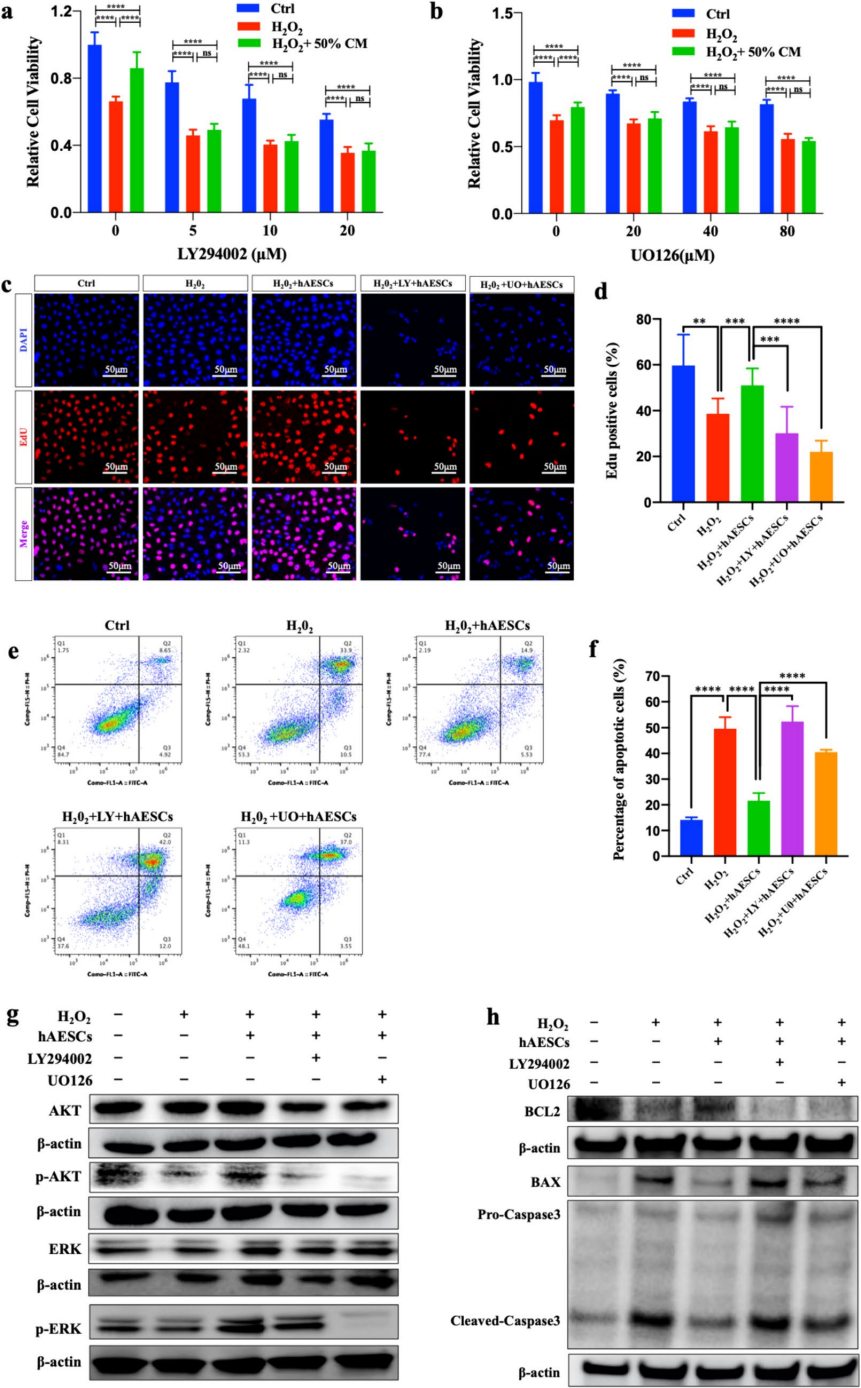
The effects of inhibitors of AKT and ERK pathway on the therapeutic effect of hAESCs-CM and hAESCs. a. The viability of KGN with or without H_2_O_2_, hAESCs-CM or LY294002 for 24 h. b. The viability of KGN with or without H_2_O_2_, hAESCs-CM or U0126 for 24 h. c. Immunofluorescence staining images of DAPI (blue) and EdU (red) of each group of KGN. Scale bar = 50 μm. d. Percent of EdU-positive cells in each group of KGN. e. Flow-cytometric analysis of Annexin V-FITC/PI of each group of KGN. f. Percent of apoptosis cells (Annexin V-FITC positive cells) in each group of KGN. g. Western blot of AKT, p-AKT, ERK, p-ERK, BCL2, BAX and β-actin in each group of KGN. h. Western blot of BCL2, BAX, Pro-Caspase3, Cleaved-Caspase3 and β-actin in each group of KGN. Data were presented as the means ± SD. ns: *P* > 0.05, **P < 0.01, ***P < 0.001, ****P < 0.0001

After co-cultured for 24 h with or without 200 μM H_2_O_2_, 50% hAESCs-CM and inhibitors under different concentrations, CCK8 detection on each group of KGN was performed. As shown in Fig. 6a and b, hAESCs-CM significantly improved the viability of H_2_O_2_-damaged KGN. However, in addition of LY294002 or U0126, the protective effect of hAESCs-CM was significantly attenuated with no difference from the H_2_O_2_-treated group. With the concentrations of LY294002 or U0126 increased, their inhibitory effects were also intensified. Thus, LY294002 and U0126 showed dose-dependent inhibitory effects on the reparative effect of hAESCs-CM on cell viability. Based on the above results, the concentrations of 20 μM of LY294002 and 80 μM of U0126 were chosen for subsequent experiments.

EdU proliferation assay was performed 48 h after co-cultivation. As shown in Fig. 6c and d, cocultured with hAESCs could significantly increase the EdU-positive cell rate of H_2_O_2_-damaged KGN. However, treatment with LY294002 or U0126 reversed the EdU-positive cell rate rescued by hAESCs. These results suggested that inhibiting the AKT or ERK pathways would block the proliferation promotion effect of hAESCs on the H_2_O_2_-damaged KGN.

Apoptosis assay was performed 24 h after co-cultivation. As shown in Fig. 6e and f, co-cultured with hAESCs markedly attenuated the damage of H_2_O_2_ on KGN apoptosis. However, the addition of LY294002 and U0126 led to a significant increase in the apoptosis rate. These results suggested that inhibiting the AKT or ERK pathways impaired the anti-apoptotic effect of hAESCs on the H_2_O_2_-damaged KGN.

Furthermore, western blot showed that LY294002 significantly inhibited the phosphorylation of AKT by hAESCs, improved the expression of BAX and Cleaved-Caspase3 and reduced the expression of BCL2. The use of U0126 markedly suppressed the phosphorylation of both AKT and ERK by hAESCs and also reversed the expression of apoptosis-related molecules (Fig. 6g and h).

## Discussion

In this study, we reported the distribution and residence of hAESCs in pZP3-induced autoimmune POI mice, and elucidated the effectiveness and potential mechanisms of hAESCs in treatment of autoimmune POI.

Most current studies utilize MSCs in POI treatment for they are the most widely used adult stem cells, widely applied in various diseases [34]. hAESCs have been found to possess similar three-lineage differentiation capability, immunomodulatory property, paracrine capability and low tumorigenicity compared to MSCs [35]. However, large-scale applications of most of MSCs are challenging owing to the invasive process and limited quantity [34]. Moreover, MSCs exhibit high heterogeneity. MSCs from different tissues express tissue-specific genes and exhibit different differentiation potentials, making them difficult to be accurately applied [36]. In contrast, hAESCs are derived from medical waste placenta, possessing advantages including indefinite tissue supply, minimal ethical concerns, and noninvasive collection procedure [37]. It is estimated that a single placenta could isolate 120 to 200 million highly pure hAESCs [38]. Furthermore, hAESCs do not express telomerase and have limited proliferative capacity, making them non-tumorigenic and safer than MSCs [20].

The mechanisms of chemotherapy-induced POI encompass DNA damage to ovarian cells and acute damage to ovarian blood vessels, resulting in ovarian cell apoptosis and stromal fibrosis [39, 40]. However, autoimmune POI is primarily instigated by inflammatory infiltration and immune dysregulation [6, 41]. Inflammation emerges as a pivotal indicator of ovarian aging which can lead to follicle depletion and impaired estradiol production and is considered to be closely related to the development of POI [42, 43]. Increased percentages of CD4 + T cells, B cells, and macrophages in the ovary were posited to correlate with ovarian aging while various cytokines, especially TNF-α, had been extensively illustrated as inducers of ovarian follicle atresia [44]. It was postulated that slight changes in tolerogenic ovary-specific antigens may prompt the generation of auto-antibodies targeting ovarian cells, thus leading to autoimmune POI [45, 46]. In addition, the attack of CD4 + cells on autologous pituitary–gonadal regulatory axis could lead to enhanced ovarian function in the early stages and subsequent development of autoimmune POI [47]. Given the distinct pathogenic mechanisms underlying autoimmune POI and chemotherapy-induced POI, it is imperative to investigate the therapeutic efficacy of hAESCs in autoimmune POI independently. Our study found that hAESCs could reduce inflammatory infiltration and reduce ovarian TNF-α expression in autoimmune POI mice, suggesting the potential immunomodulatory effect of hAESCs. Moreover, it was reported that hAESCs could downregulate the ratios of Th17/Treg cells and improve the cytokine environment in the models of Hashimoto's thyroiditis and Systemic lupus erythematosus [22]. Similarly, in the autoimmune encephalomyelitis model, hAESCs were also found to inhibit inflammatory infiltration and regulate the cytokine environment and the balance of immune cells [48]. In addition, recently, it was found that MSCs enriched in the liver after intravenous injection could induce the liver to secrete insulin like growth factor 1 to reduce inflammation in colitis [49]. However, whether hAESCs could also play an anti-inflammatory role in autoimmune POI mice by inducing extra-ovarian organs to secrete cytokines or regulating immune cells requires further study.

Monitoring the distribution of stem cells after transplantation contributed to gain further insights into the potential therapeutic mechanisms of stem cell treatment. Research on different disease models indicated that intravenously injected hAESCs could migrate to the injury sites and differentiate into appropriate cells [50]. Wang et al. proposed that hAESCs could differentiate into FSHR-positive GCs in the ovarian follicles of chemotherapy-induced POI mice model [23]. However, Stilley et al. [51] showed that FSHR was expressed not only in hAESCs but also in ovarian tissues. Therefore, rather than utilizing differentiation potential, hAESCs were more likely to repair damage through other potential pathways. Additionally, Srinivasan et al. demonstrated that intravenous injection of hAESCs resulted in 5–15% of hAESCs enriched in the liver and 40% of hAESCs enriched in the lungs [52] which was consistent with our results. A study on intravenous injection of bone marrow mesenchymal stem cells (BMSCs) for the treatment of cisplatin-induced POI model showed that BMSCs were mainly distributed in the ovary hilum and medulla, as well as the sheath of the ovarian cortex, instead of follicles and corpus luteum [53]. However, our work found that the transplanted hAESCs were mainly distributed in the GC layer of follicles in the ovary of autoimmune POI model which might related to the injury chemotaxis of hAESCs and required further research. Ovarian GCs are one of the most important components of the ovarian follicles. They provide nutrients and hormonal support through the zona pellucida, which is indispensable for the maturation of oocytes [54]. Follicle atresia caused by GCs apoptosis is the main process of follicle and oocyte loss in mammalian ovaries, and is also a key mechanism of POI [55, 56]. And restoration of GCs’ function is considered an important mechanism for restoring endocrine function in autoimmune POI [57].Several studies confirmed that hAESCs-CM and hAESCs-derived exosomes could improve ovarian function in chemotherapy-induced POI mice [24–27]. Cytokine array showed that hAESCs could secrete a variety of cytokines, such as epidermal growth factor, hepatocyte growth factor and FGF2 [27]. In our study, we found that hAESCs could significantly alleviate the apoptosis of GCs in autoimmune POI, reduce the damage of mitochondria and endoplasmic reticulum in GCs. In addition, hAESCs or hAESCs-CM cocultivation could reduce apoptosis and rescue cell viability and proliferation of H_2_O_2_-damaged KGN. These results indicated that hAESCs could reduce apoptosis and promote proliferation of GCs through the paracrine pathway, thereby strengthening the support for oocytes and follicle development and reducing follicle atresia and oocyte loss. Some have found that MSCs could improve the fertility of POI mice by improving oocyte quality in a chemotherapy-induced POI mouse model [20, 21]. Whether hAESCs can exert similar effects on oocytes in autoimmune POI requires further investigation.

One of the principal beliefs in reproductive biology is that that the entire oocyte pool is endowed at birth or soon after birth most mammalian females [58]. Our study showed that 4 weeks after hAESCs transplantation, the number of follicles in the hAESCs group was greater than that in the POI group, including the number of primordial follicles. Rhim et al. observed a loss of large follicles, rather than primordial follicles, in pZP3-induced autoimmune POI mice 21 days after the initial immunization [59]. However, the number of primordial follicles decreased significantly 70 days after the initial immunization [60]. Therefore, we speculated that hAESCs treatment, which was administered 28 days after the initial immunization, inhibited the reduction of primordial follicles in POI mice. However, recent studies have indicated the presence of oogonial stem cells (OSCs) in the postnatal ovaries of humans, mice, and rats [58]. Diksha et al. found that OSCs in adult mice ovaries could undergo differentiation, neo-oogenesis and form follicles under physiological conditions [61]. In addition, exogenous interference may lead to dysfunction of OSCs, ultimately leading to POI [62]. Further research is needed to determine whether autoimmune POI could affect the function of OSCs and whether hAESCs can treat POI through improving OSCs function.

Through transcriptome sequencing and further KEGG and PPI analysis, we screened out several pathways, including AKT and ERK pathways, and some hub genes that might be involved in the mechanism of hAESCs treatment. It was reported that the proliferation of GCs required the co-activation of the AKT pathway and the ERK pathway, while inhibiting the phosphorylation of AKT and ERK would inhibit proliferation and promote apoptosis of mice GCs [63, 64]. AKT pathway has been recognized to mediate the activation of primordial follicles [65]. Moreover, in vitro follicle activation via stimulating AKT pathway had been used to treat infertility in POI in the clinic with live births reported [65]. ERK pathway is involved in cytoplasmic maturation and meiosis of oocytes [66]. Activation of the ERK pathway can enhance oocyte quality and mitochondrial function [66]. Conditional knockout of ERK in GCs would lead to abnormal oocyte development, ovulation failure and infertility in mice, which suggested the importance of activation of ERK in GCs [67]. Our study also confirmed that hAESCs could help KGN resist H_2_O_2_ damage by activating the AKT and ERK pathway.

Here, we comprehensively demonstrated the therapeutic effect of hAESCs in the treatment of autoimmune POI mice, providing the possibility for clinical application of hAESCs in the treatment of autoimmune POI. Through tracking up to 4 weeks, we clarified for the first time the distribution pattern of intravenously injected hAESCs in autoimmune POI mice, which provided data support for further optimizing the method, dosage and interval of hAESCs administration. Previous studies have used proteomics analysis [27, 68] and miRNA sequencing [68, 69] to analyze the cytokines and exosomes secreted by hAESCs. We directly conducted transcriptomic analysis on the ovaries of hAESCs-treated autoimmune POI mice, further exploring the mechanism of hAESCs treatment. Taken together, these findings may facilitate the development of new hAESCs-based therapeutic approaches to autoimmune POI.

There are some limitations in the present study. The molecular mechanisms in animal model have only been superficially explored. In addition, the impact of hAESCs on other target cells in autoimmune POI, such as oocytes and immune cells, remains to be ascertained and the roles of other pathway and hub genes screened out by KEGG and PPI analysis warrant further exploration.

Next, other issues still have to be resolved in the application of hAESCs, such as optimizing the administration mode of hAESCs, long-term safety of hAESCs treatment, pretreatment of hAESCs and development of hAESCs-based cell-free drugs.

## Conclusion

In summary, we found that tail vein injected hAESCs could reside in autoimmune POI mice for at least 4 weeks. hAESCs had significant therapeutic effects on impaired follicular development, ovarian function and fertility in autoimmune POI mice. hAESCs could promote the proliferation and reduce the apoptosis of GCs via activating the AKT and ERK pathways through the paracrine mechanism, which might be the key mechanism for hAESCs treatment.

### Supplementary Information

Below is the link to the electronic supplementary material.

Supplementary file1 (PDF 72 KB)

## Data Availability

The datasets used and/or analyzed during the current study are available from the corresponding author on reasonable request.
