# Supplementary material for: Human Amniotic Epithelial Stem Cells Alleviate Autoimmune Premature Ovarian Insufficiency in Mice by Targeting Granulosa Cells via AKT/ERK Pathways
Source: Stem Cell Rev Rep. 2024 Jun 4;20(6):1618–35. doi: 10.1007/s12015-024-10745-z (PMC11319531; doi:10.1007/s12015-024-10745-z)
Supplement: Supplementary file 1 — Supplementary file1 (PDF 72 KB) [file 12015_2024_10745_MOESM1_ESM.pdf]

Additional file 1: Table S1 The qRT-PCR primer sequences

Gene	Primer	Sequence (5' to 3)
Gdf9	Forward	GTCACCTCTACAATACCGTCCG
	Reverse	CACCCGGTCCAGGTTAAACA
Hmox1	Forward	AAGCCGAGAATGCTGAGTTCA
	Reverse	GCCGTGTAGATATGGTACAAGGA
Amh	Forward	CCACACCTCTCTCCACTGGTA
	Reverse	GGCACAAAGGTTCAGGGGG
Fgf2	Forward	GCGACCCACACGTCAAACTA
	Reverse	TCCCTTGATAGACACAACTCCTC
P21	Forward	CCTGGTGATGTCCGACCTG
	Reverse	CCATGAGCGCATCGCAATC
Gapdh	Forward	CAGGAGGCATTGCTGATGAT
	Reverse	GAAGGCTGGGGCTCATTT
